# Journal de la Physiologie de l’Homme et des Animaux

**Published:** 1859-10

**Authors:** 


ART. VII.—*Journal de la Physiologie de l'Homme et des Animaux.*

We have received, through the politeness of B. Westermann & Co., the sixth number of this valuable physiological journal. This has now been published for a year and a half, and is undoubtedly a success in a business, as it is in a professional, point of view. This number contains ten valuable original articles; in addition to which we have translations, extracts from periodical publications, &c., making one of the most valuable numbers which has been issued. That a journal so expensively gotten up, and devoted to the single branch of physiology, should succeed, speaks well for the rapid advancement of this department of medical science.

